# Profile of people with hypertension in Nairobi’s slums: a descriptive study

**DOI:** 10.1186/s12992-015-0112-1

**Published:** 2015-06-27

**Authors:** Annelieke Hulzebosch, Steven van de Vijver, Samuel O. Oti, Thaddaeus Egondi, Catherine Kyobutungi

**Affiliations:** Utrecht University, Ameland 195 3524 AN, Utrecht, Netherlands; Department of Global Health, Academic Medical Center, University of Amsterdam, Nairobi, Kenya; African Population and Health Research Center, Nairobi, Kenya

**Keywords:** Hypertension, Slums, Sub-Saharan Africa, Overweight, Awareness, Drug coherence, Diabetes

## Abstract

**Background:**

Cardiovascular disease (CVD) is a rising health burden among the world’s poor with hypertension as the main risk factor. In sub-Saharan Africa, hypertension is increasingly affecting the urban population of which a substantial part lives in slums. This study aims to give insight into the profile of patients with hypertension living in slums of Nairobi, Kenya.

**Methods:**

Sociodemographic and anthropometric data as well as clinical measurements including BP from 440 adults with hypertension aged 35 years and above living in Korogocho, a slum on the eastern side of Nairobi, Kenya, will be collected at baseline and at the first clinic visit.

**Conclusion:**

The study population showed high prevalence of overweight and abdominal obesity as well as behavioral risk factors such as smoking, alcohol and a low vegetable and fruit intake. Furthermore, the majority of hypertensive patients do not take anti-hypertensive medication and the ones who do show little adherence.

**Trial registration:**

Current controlled trials ISRCTN84424579.

## Background

In 2000, nearly one billion of the world’s population (over 25 % at that time) had hypertension and this is expected to increase to almost 30 % by 2025 [1]. Hypertension is the single most important risk factor for cardiovascular disease (CVD) [2, 3], the leading cause of death worldwide [3]. The World Health Organization estimated that CVD caused 13.4 million deaths in 2008, 80 % of which occurred in developing countries [4, 5].

In sub-Saharan Africa (SSA), the epidemiological transition of diseases causing mortality from predominantly infectious diseases to non-communicable diseases (NCDs) like CVD is attributable largely to urbanization and industrialization [6]. The changes in lifestyle associated with this transition may lead to an increase in the occurrence of behavioral risk factors for CVD such as smoking, physical inactivity and an unhealthy diet. Increases in mean blood pressure (BP) have already been observed in SSA. In Kenya for example, the average systolic BP has risen from 127 in 1990 to 132 mmHg in 2010 [7]. The prevalence of risk factors for CVD including hypertension is higher in urban than in rural areas [8, 9]. It is expected that the urban population in Africa will increase by half in the next decades [10]. The urban population growth rate in Nairobi is 4.2 %, which is almost double the national growth rate of 2.4 % [11]. Nearly 60 % of the urban population in Nairobi lives in slums or slum-like conditions [12, 13]. Slums are typically characterized by poor living conditions with limited access to quality healthcare [14]. The prevalence of risk factors for CVD is high in slums and the associated psychosocial burden of insecurity, violence and stress may cause an increased risk of CVD [15]. Therefore a large part of the CVD burden is on the urban poor, who may not have the financial resources or adequate health literacy to adopt healthier lifestyles to adopt preventive measures [16, 17].

Despite the large extent of the epidemic, there is a paucity of documentation on the profile of the growing group of urban poor people with hypertension in SSA. This descriptive study aims to give an insight into patients with hypertension in a slum in Nairobi. What are the characteristics of these people, their behavioral risk factors, medication use and comorbidities?

## Methods

### Study population and design

The study was conducted in Korogocho, a large slum with approximately 72.000 residents in the outskirts of Nairobi, the capital city of Kenya. Korogocho is under surveillance as part of the Nairobi Urban Health and Demographic Surveillance System (NUHDSS). The NUHDSS is operated by the African Population and Health Research Center (APHRC), a regional research institution. Details of the operation of the NUHDSS have been published elsewhere [18].

Data from 440 individuals were collected between August 2012 and December 2013. The data-collection was done as part of a published cross-sectional survey called the SCALE UP project, which aimed to develop and introduce a cost-effective and scalable CVD prevention model among the urban poor [19]. The goal of the prevention model was to raise awareness of hypertension through mass screening of adult slum residents aged 35 years and older, to improve access to treatment and promote adherence to medication. This paper examines adults with hypertension detected through the SCALE UP study and referred to local primary health care facility for treatment and follow-up.

As part of the SCALE UP study, 40 interviewers and 40 community health workers (CHWs) trained and employed by APHRC performed household visits to screen participants for hypertension and risk factors. The interviewers administered the questionnaire in the local lingua franca – Kiswahili. This questionnaire was adapted from the WHO STEPs instrument and gathered information on demographics, education level, wealth and behavioral risk factors [20]. After the questionnaires were administered, the CHWs proceeded to take anthropometric measurements from all consenting participants including weight, height and waist circumference. While an elevated BMI is a well-established contributor to the etiology of CVD, studies have shown that abdominal obesity are more closely related to CVD morbidity and mortality than BMI alone [20]. Therefore, both measures have been included in this study. All measurements were taken in line with the STEPS protocol [21]. Stage one hypertension was defined as mean systolic blood pressure (SBP) at least 140 mmHg or diastolic blood pressure (DBP) at least 90 mmHg, or self-reported previous diagnosis and current use of antihypertensive medication. Stage two hypertension was defined as mean SBP at least 160 mmHg or DBP at least 100 mmHg [2]. Mean arterial pressure was diastolic pressure plus one third of systolic BP. Treatment control was defined as BP of less than 140/90 mmHg for those participants currently taking anti-hypertensive medication. Participants with hypertension detected through the household visits were referred to a local primary health clinic known as Provide International, where they were interviewed again trained community health workers for risk re-assessment purposes. Participants were then assessed by clinical officers who confirmed their diagnoses. Those confirmed with hypertension and who provided informed consent became subjects of this current study. Overall, three levels of information were collected from participants; a questionnaire to gather information about history of hypertension and comorbidities, basic anthropometric and clinical measurements and a random blood glucose test.

Average monthly income was categorized into four groups, where 1000 KES equals $11.10. Alcohol consumption was classified as low (<1 standard unit of alcohol per week), moderate (1–14 per week for males and 1–7 per week for females) and high (>14 per week for males and >7 per week for females). Adequate physical activity was defined as more than 150 min of moderate intensity activity or more than 75 min vigorous intensity activity per week [22, 23]. Adequate fruit and vegetable intake was defined as 5 servings of fruit or vegetables per day, using a serving size of 80 g and the daily minimum of 400 g [24–26].

Field supervisors verified the data quality. Questionnaires that did not meet the standards were corrected in the field. The completed questionnaires were then transported to a central location for double-data entry using MySQL data entry screens with Microsoft Access database backend.

### Statistical analysis

Frequency distributions of sociodemographic, behavioral and clinical characteristics of study subjects by gender were examined. Continuous variables were expressed as means with standard errors of means. Categorical variables were expressed as numbers with percentages. For those variables where over 10 % of results were missing, ‘missing’ was listed as a category and thus percentages adjusted accordingly.

Association of the study participants’ sociodemographic and behavioral risk factors by sex was conducted using chi square tests for significance. A value of *P* < 0.05 was considered significant. All analyses were performed with STATA 12 (Stata Corp, College Station, Texas).

### Ethical approval

The study protocol was approved by the Kenya Medical Research Institute (KEMRI)/National Ethical Review Committee (NON-SSC Protocol No.339).

## Results

There were 256 women (58 %) and 184 men (42 %) in the study. It is noteworthy that although a total of 976 were referred from the screening during the household visits, only 440 (46 %) who attended the local primary clinic subsequently as at the time of conducting this analysis were included.

Table 1 shows sociodemographic variables as well as behavioral risk factors. Men were significantly more educated than women and reported a higher average monthly income. Gender disparities were also observed in CVD risk factors, significantly more men smoked and consumed alcohol. There was no significant difference in reported physical activity and daily fruit and vegetable intake between genders. Overall individuals demonstrated a high level of physical activity but a very low level of fruit and vegetable intake. As can be seen in Table 2, women had a higher BMI with 63 % being overweight or obese. Men had a significantly larger waist circumference; almost half of them fit the criteria for abdominal obesity. The mean SBP was 149.2 mmHg and the mean DBP was 95.2 mmHg. Women had a lower SBP than men. Over 40 % of individuals fit the criteria for stage two hypertension. Table 3 shows the history of the participants’ hypertension. Almost 70 % were diagnosed in the last two years and 68 % were diagnosed through a CHW visiting their home as part of the SCALE UP study. Only a quarter of the participants with hypertension ever used medication for their condition. Of the people who did, the majority (78 %) used the medication daily. The reported total costs of anti-hypertensive medication vary but over 75 % spent more than 100 KES per month (about $1.15). Treatment control was 53.8 % (95 % CI 34.7–73.0). Almost half of the medication users are currently not taking the medication anymore. Over a third of patients felt better and therefore believed they did not need the medication anymore, and almost half of the group reported they stopped using the anti-hypertensive drugs because they could no longer afford it. Overall 10 % of the patients with hypertension were found to have diabetes. Only 6.8 % reported to be aware of this condition prior to the CHW’s home visit. Table 4 shows other reported comorbidities.

**Table 1:** Description of study participants by gender

	All (*N* = 440)			Women (*N* = 256)			Men (*N* = 184)		
	*N*	%	95 % CI	*N*	%	95 % CI	*N*	%	95 % CI
**Age (years)**									
<45	67	15.2	12.2–18.9	41	16.0	12.0–21.1	26	14.1	9.8–20.0
45–54	154	35.0	30.7–39.6	92	35.9	30.3–42.0	62	33.7	27.2–40.9
55–64	102	23.2	19.5–27.4	51	19.9	15.5–25.3	51	27.7	21.7–34.7
65–74	78	17.7	14.4–21.6	46	18.0	13.7–23.2	32	17.4	12.6–23.6
≥75	39	8.9	6.5–11.9	26	10.2	7.0–14.5	13	7.1	4.1–11.8
**Education** ***P < 0,01***									
Not finished primary school	99	22.5		66	25.8		38	17.9	
Primary school	171	38.9		89	34.8		82	44.6	
Secondary school and higher	54	12.3		16	6.3		33	20.7	
Missing	116	26.4		85	33.2		31	16.8	
**Marital status** ***P < 0,01***									
Currently married	269	61.3		108	42.2		161	88.0	
Never married	30	6.8		23	9.0		7	3.8	
Widowed	89	20.3		80	31.3		9	4.9	
Divorced/separated	51	11.6		45	17.6		6	3.3	
**Monthly income** ***P < 0,01***									
<1000 KES	37	8.4		29	11.3		8	4.3	
1000–4999 KES	180	40.9		119	46.5		61	33.2	
5000–9999 KES	88	20.0		31	12.1		57	31.0	
≥10 000 KES	23	5.2		5	2.0		18	9.8	
Missing	112	25.5		72	28.1		40	21.7	
**Smoking status** ***P < 0,01***									
Current smoker	37	8.4		5	2.0		32	17.5	
Previous smoker	52	11.8		18	7.0		34	18.6	
Non-smoker	350	79.7		233	91.0		117	63.9	
**Alcohol consumption** ***P < 0,01***									
Low	373	84.8		237	92.6		136	73.9	
Moderate	30	6.8		9	3.5		21	11.4	
High	37	8.4		10	3.9		27	14.7	
**Physical activity** ***P = 0.17***									
Adequate	401	91.8		231	90.2		170	93.9	
Inadequate	36	8.2		25	9.8		11	6.1	
**Daily fruit and vegetable intake** ***P = 0.12***									
Adequate	76	17.3		49	19.1		27	14.7	
Inadequate	299	68.0		176	68.8		123	66.8	
Missing	65	14.8		31	12.1		34	18.5	

Missing data: marital status 1, smoking status 1, physical activity 3. *P* values for difference between genders derived using chi-squared test

**Table 2:** Anthropometric and biochemical measurements

	All (*N* = 440)			Women (*N* = 256)			Men (*N* = 184)		
	*N*	%	95 % CI	*N*	%	95 % CI	*N*	%	95 % CI
**BMI** ***P < 0.01***									
Underweight (<18.5)	26	6.0	4.1–8.7	13	5.2	3.0–8.8	12	7.2	4.2–12.0
Normal weight (18.5–24.9)	184	42.8	38.2–47.5	79	31.7	26.2–37.8	105	58	50.7–65.0
Overweight (25–29.9)	130	30.2	26.1–34.8	88	35.3	29.6–41.5	42	23.2	17.6–29.9
Obese (≥30)	90	20.9	17.3–25.1	69	27.7	22.5–33.6	21	11.6	7.7–17.2
**Waist circumference** ***P < 0.01***									
Normal	293	67.8	63.3–72.1	200	79.7	74.2–84.2	93	51.4	44.1–58.6
Abdominal obesity	139	32.2	27.9–36.7	51	20.3	15.8–25.8	88	48.6	41.4–55.9
**Diabetes mellitus**	41	10.0	7.5–13.4	26	11.0	7.6–15.6	15	8.8	5.3–14.1
**Blood pressure**									
Mean SBP	149.2	147.1–151.3		147.3	144.4–150.1		151.9	148.7–155.1	
Mean DBP	95.2	94.0–96.4		95.5	94.0–97.0		94.8	92.9–96.7	
Mean arterial pressure	145.0	143.2–146.7		144.6	142.4–146.9		145.4	142.7–148.2	
Stage two hypertension	177	40.2	35.7–44.9	100	39.1	33.3–45.2	77	41.8	34.9–49.1

Missing data: BMI 10, waist circumference 8, diabetes mellitus 32. *P* values for difference between genders derived using chi-squared test and *t*-test in the case of blood pressure

**Table 3:** History of hypertension

	All (*N* = 440)		
	*N*	%	95 % CI
**Time since diagnosis (years)**			
<1	14	3.2	1.9–5.3
1–2	285	64.8	60.2–69.1
2–5	56	12.7	9.9–16.2
5–10	39	8.9	6.5–11.9
>10	46	10.5	7.9–13.7
**Place of diagnosis**			
CHW visit	298	68.5	
Private healthcare facility	92	21.1	
Public healthcare facility	45	10.3	
**Ever used anti-hypertensive drugs**			
No	336	76.5	
Yes	103	23.5	
**Medication use**			
Daily	80	77.7	
On most days	10	9.7	
On some days	7	6.8	
When I felt bad	6	5.8	
**Total cost of anti-hypertensive drugs per month**			
No cost, free	13	12.6	
<100 KES	11	10.7	
100–299 KES	30	29.1	
300–499 KES	28	27.2	
≥500 KES	21	20.4	
**Currently taking the anti-hypertensive drugs**			
Yes	54	52.4	
No	49	47.6	
**Reason for stopping the anti-hypertensive drugs**			
Could no longer afford the medication	25	51.0	
I felt better, did not need further treatment	15	30.6	
Other	9	18.4	

Missing data: place of diagnosis 2, ever used anti-hypertensive drugs 1

**Table 4:** Self reported history of comorbidities

	All (*N* = 440)			Women (*N* = 256)			Men (*N* = 184)		
	*N*	%	95 % CI	*N*	%	95 % CI	*N*	%	95 % CI
Diabetes	30	6.8	4.8–9.6	23	9.0	6.0–13.2	7	3.8	1.8–7.8
Stroke	7	1.6	0.8–3.3	3	1.2	0.4–3.6	4	2.2	0.8–5.7
Angina	5	1.1	0.5–2.7	3	1.2	0.4–3.6	2	1.1	0.3–4.3

Missing data: diabetes, stroke and angina 1

## Discussion

This study describes the profile of hypertensive patients living in an informal settlement in Kenya. Compared to a recent population wide survey in Korogocho, our study participants smoke and consume more alcohol, and there is more obesity and less fruit and vegetable intake [15]. The differences could be explained by the type of study participants, our study only included hypertensive patients aged 35 years and older whereas the previous survey included a representative urban slum population of adults aged 18 years and older. So compared to a largely healthy population, patients with hypertension will expectedly show different characteristics. For example, the percentage of men in our study with abdominal obesity (48.6 %) is very high compared to the aforementioned survey in the same slum where only 11.6 % of men fit the criteria.

Our study shows a higher rate of participants classifying as overweight or obese than a recent study on overweight in Korogocho that found overweight and obesity rates similar to that of a national survey; around 40 % in women and 17 % in men [27, 28]. Studies from Tanzania and Cameroon show roughly the same gender disparities in prevalence of excess bodyweight [29, 30]. In our study we have found overweight and obesity prevalence to be much higher at 63 % and 34.8 % for women and men respectively, but again, our study sample consists of selected individuals with known hypertension. The high proportion of overweight and obesity specifically among women could be explained by different socio-cultural factors such as gender specific patterns of work activities and cultural standards of physical attractiveness [31].

Currently the estimated prevalence of diabetes in Africa is 1–3 % in rural areas and 5–6 % in urban SSA, but country reports have varied widely [32]. The prevalence of diabetes is likely to be higher in people with hypertension than at a population level. A study in Nigeria found that 4.6 % of hypertensive patients had diabetes [33]. Our study found this number to be over twice as high (10 %).

Evidence shows that CVD risk factors occur predominantly in clusters [34]. People with diabetes are more likely to also suffer from hypertension and/or dyslipidemia. This is likely to also occur in our study population, given the high obesity and abdominal obesity prevalence suggesting a strong influence of metabolic syndrome in this population.

### Recommendations

There should be more attention paid to the large and growing epidemic of CVD in SSA and a joint effort to stop it. Policy makers and health providers need to work together. This paper shows that weight control is an important priority in both men and women and that cost of medication plays a major role in compliance. A possible solution could be a community health insurance or drug revolving funds [35].

Levels of health literacy have to increase in order for people to change their lifestyle, get diagnosed or comply with therapy. The WHO developed”best buys” for policy makers to tackle the main non-communicable diseases. These “best buys” state that providing counseling and multi-drug therapy for people with high risk of CVD can be used together with population-level interventions like taxes on tobacco to decentivize smoking, legislation to promote reduced salt foods for mitigating hypertension and improving the public awareness of the role of diet and physical activity on health through mass media. These interventions have been found to have a significant impact and be highly cost-effective. The implementation of these initiatives in a slum context is urgently needed.

### Strengths and limitations of the study

To the best of our knowledge this is the first study on the profile of hypertensive patients in a Kenyan urban informal settlement. Our study adds to the limited body of evidence on hypertension in Kenya and gives an insight to the patients’ living standards and their rationale. This study has several limitations. One is the self-reporting of patients’ wealth, education, behavioral risk factors (smoking, alcohol misuse, diet and physical activity), comorbidities and use of medication. Self-reporting can lead to inaccurate reporting due to lack of awareness, misinterpretation of questions, or concern for judgement and affect our study conclusions. Additionally, our study did not capture over 50 % of patients during household screening visits or clinic visits. Follow up studies in this sub-population of patients would be very helpful. Despite these limitations, this study provides important data regarding the profile of patients with hypertension in an African urban slum.

## Conclusions

The study population showed high prevalence of overweight and abdominal obesity as well as behavioral risk factors such as smoking, alcohol and a low vegetable and fruit intake. Furthermore, the vast majority of hypertensive patients do not take anti-hypertensive medication and the ones who do show little adherence.

## Abbreviations

CVD: Cardiovascular disease
SSA: Sub-Saharan Africa
NCDs: Non-communicable diseases
BP: Blood pressure
NUHDSS: Nairobi Urban Health and Demographic Surveillance System
CHW: Community health worker
SBP: Systolic blood pressure
DBP: Diastolic blood pressure
KEMRI: Kenya medical research institute
